# Oxaliplatin plus irinotecan vs irinotecan as second-line treatment in pancreatic cancer patients: a randomized–controlled open-label Phase II study

**DOI:** 10.1093/gastro/goac088

**Published:** 2023-02-03

**Authors:** Hangyu Zhang, Zhou Tong, Lulu Liu, Qihan Fu, Xudong Zhu, Xiaomeng Dai, Xuanwen Bao, Weijia Fang, Yi Zheng, Peng Zhao

**Affiliations:** Department of Medical Oncology, School of Medicine, First Affiliated Hospital, Zhejiang University, Hangzhou, Zhejiang, P. R. China; Department of Medical Oncology, School of Medicine, First Affiliated Hospital, Zhejiang University, Hangzhou, Zhejiang, P. R. China; Department of Medical Oncology, School of Medicine, First Affiliated Hospital, Zhejiang University, Hangzhou, Zhejiang, P. R. China; Department of Medical Oncology, School of Medicine, First Affiliated Hospital, Zhejiang University, Hangzhou, Zhejiang, P. R. China; Department of Medical Oncology, School of Medicine, First Affiliated Hospital, Zhejiang University, Hangzhou, Zhejiang, P. R. China; Department of Medical Oncology, School of Medicine, First Affiliated Hospital, Zhejiang University, Hangzhou, Zhejiang, P. R. China; Department of Medical Oncology, School of Medicine, First Affiliated Hospital, Zhejiang University, Hangzhou, Zhejiang, P. R. China; Department of Medical Oncology, School of Medicine, First Affiliated Hospital, Zhejiang University, Hangzhou, Zhejiang, P. R. China; Department of Medical Oncology, School of Medicine, First Affiliated Hospital, Zhejiang University, Hangzhou, Zhejiang, P. R. China; Department of Medical Oncology, School of Medicine, First Affiliated Hospital, Zhejiang University, Hangzhou, Zhejiang, P. R. China

**Keywords:** pancreatic cancer, second-line chemotherapy, randomized control trial, irinotecan, IROX

## Abstract

**Background:**

Limited second-line therapeutic options are available for metastasis pancreatic cancer (mPC). We aimed to explore the efficacy and safety of oxaliplatin plus irinotecan (IROX) in mPC patients.

**Methods:**

This is an open-label, Phase 2, randomized study of mPC patients (aged 18–75 years) who failed when using gemcitabine plus S-1 as first-line therapy. Block randomization with a block size of four was used to randomly assign patients (1:1) between October 2015 and December 2017 to receive either IROX (oxaliplatin 85 mg/m^2^ and irinotecan 160 mg/m^2^) or irinotecan monotherapy (irinotecan 180 mg/m^2^) until disease progression, unacceptable adverse events, or consent withdrawal. The primary end point was overall survival, and the secondary end points were progression-free survival, overall response rate, and adverse event rate.

**Results:**

A total of 74 patients were enrolled in this study, including 44 males and 30 females, with an average age of 61 years. The median overall survival was 10.2 and 6.7 months (adjusted hazard ratio [HR], 0.7; 95% confidence interval [CI], 0.4–1.2; *P *=* *0.20) and the median progression-free survival was 5.1 and 2.3 months (adjusted HR, 0.4; 95% CI, 0.2–0.6; *P < *0.01) in the IROX group and irinotecan group, respectively. The overall response rates were 18.4% (7/38) in the IROX group and 5.5% (2/36) in the irinotecan group (*P *=* *0.06). Grade 3–4 adverse events occurred in 34% (13/38) of patients in the IROX group and 19% (7/36) of patients in the irinotecan group (*P *=* *0.15).

**Conclusions:**

IROX had no significant survival benefit over irinotecan monotherapy in our study. However, IROX reduced the risk of disease progression by 60%, with acceptable toxicity.

## Introduction

Pancreatic cancer (PC) is the fourth leading cause of cancer death, and the incidence rises by ∼1% per year in both males and females [1]. The 5-year survival rate of PC patients, ∼10% for all stages combined, is poor, which is attributed to its poor biological behavior, difficulty in early detection, and lack of targeted therapy and individual therapy options [2]. The median survival time of patients with metastatic pancreatic cancer (mPC) usually ranges from 6 to 11 months [3].

Although various new drugs, including immune checkpoint inhibitors, have been tested in the treatment of PC, they ultimately failed in most studies. Even high microsatellite instability (MSI-H) mPC derives very limited benefit from immunotherapy [4]. The standard first-line therapies are gemcitabine-based chemotherapy and the FOLFIRINOX regimen for mPC patients with good performance status. The GEST study showed that the progression-free survival (PFS) in the gemcitabine plus S-1 (GS) group was significantly longer than that in the gemcitabine monotherapy group (median PFS, 5.7 vs 4.1 months, *P *<* *0.001) and showed an overall survival (OS) advantage trend (median OS, 10.1 vs 8.8 months) [5]. Thus, GS is currently one of the first-line treatment regimens recommended by the Chinese Society of Clinical Oncology [6].

Regarding second-line treatments, there are no guideline-preferred treatments; other recommended regimens include liposomal irinotecan plus 5-fluorouracil, FOLFIRI and OFF [7, 8]. Although the Food and Drug Administration of the USA approved nanoliposomal irinotecan as a second-line option [9], it is not available in many countries including China. An appropriate second-line treatment is urgently needed by a large proportion of patients who progressed with GS or gemcitabine combined with capecitabine as an adjuvant or first-line therapy regimen. However, Asian patients are reported to experience more frequent hematologic toxicities than Western patients when treated with FOLFIRINOX [10]; a previous study suggested that a response to repeated use of fluorouracil is highly unlikely [11]. Therefore, we performed this randomized–controlled trial to explore the superiority of oxaliplatin plus irinotecan (IROX) over irinotecan monotherapy as a second-line option for mPC patients.

## Patients and methods

### Study population

Our study was reviewed and approved by the Ethics Committee of the First Affiliated Hospital of Zhejiang University School of Medicine (Hangzhou, China). Details about the study are available on ClinicalTrials.gov (NCT02558868). Written informed consents were signed by all patients. The inclusion criteria were as follows: patients with histologically confirmed pancreatic ductal adenocarcinoma who had progressed after first-line GS therapy or had metastasis within 6 months after adjuvant GS treatment; age ranging from 18 to 75 years; Karnofsky performance status of ≥70%; adequate renal, hepatic, and bone marrow function; and at least one measurable site on computed tomography or magnetic resonance imaging. The exclusion criteria are: prior or concurrent malignant disease within 5 years; prior use of study drugs; the presence of poorly controlled hypertension, diabetes, serious cardiovascular disease, or other chronic diseases; cardiovascular- or cerebrovascular-related events within 4 weeks before randomization; peripheral neurotoxicity more than Grade 2; or pregnancy or breastfeeding.

### Study design

This prospective, open-label, Phase II trial assigned patients in a 1:1 ratio to the IROX group or irinotecan monotherapy group through block randomization with a block size of four. The IROX regimen contained both oxaliplatin (85 mg/m^2^, 120 min) and irinotecan (160 mg/m^2^, 120 min) every 2 weeks. The irinotecan monotherapy regimen involved irinotecan (180 mg/m^2^, 120 min) every 2 weeks. When Grade 3–4 adverse events (AEs) according to the Common Terminology Criteria for Adverse Events (CTCAE) occurred, the dose of chemotherapy was reduced by 20%. Treatment was continued until disease progression or adverse reaction intolerance occurred or until informed consent was withdrawn for any reason.

### Outcome measurement

All patients who received at least one cycle of chemotherapy were included in the analysis. We collected demographic data, tumor-site information, serum levels of carbohydrate antigen 19–9 (CA19-9), and surgery information at baseline, and dynamically recorded tumor characteristics.

The primary end point of this study was OS and the secondary end points were PFS, overall response rate (ORR), and AE rate according to CTCAE version 4.0. Tumor response was regularly assessed every 6 weeks and several patients with clinical progression were evaluated earlier according to the Response Evaluation Criteria in Solid Tumors (RECIST) version 1.1 [12]. An early CA19-9 response was defined as a decrease in the serum CA19-9 level from baseline after one cycle of treatment. PFS was defined as the time from the first treatment after randomization to the date of progression or the date of patient death. ORR was calculated as the ratio of patients with a response (partial response [PR] and complete response [CR]) to all patients. The disease control rate (DCR) was calculated as the proportion of patients with stable disease (SD), PR, and CR out of all patients. For patients lost to follow-up, the last observation time was censored.

### Statistical analysis

We analysed the intention-to-treat population (excluding patients who withdrew prior to treatment and had no recorded treatment-related data). A one-sided log-rank test was performed to calculate the sample size of 80 patients (40 per group), with 80% power of a one-sided type I error rate of 0.1. PFS, and OS values were estimated from Kaplan–Meier curves and analysed by using the log-rank test to compare the cumulative survival durations. Univariate analysis was first performed between each variable and PFS/OS, and multivariate analysis was then performed with stepwise Cox proportional hazards regression modeling for variables of interest with a *P*-value of <0.10. Hazard ratios (HRs) with 95% confidence intervals (CIs) were used to express the relative risks. *P *<* *0.05 was considered statistically significant for all tests. IBM SPSS Statistics (Version 20.0; IBM Corp, New York, USA) was used for the analyses. Figures were created using GraphPad Prism 7 (GraphPad Software, CA, USA).

## Results

### Patient characteristics

**Figure 1.**
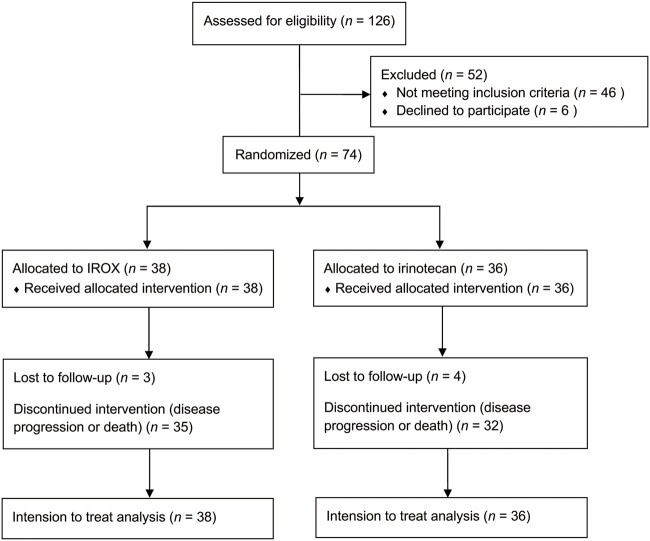
CONSORT flow diagram.

Between October 2015 and December 2017, a total of 126 patients were screened, of whom 46 did not meet the inclusion criteria because their first-line treatment was not the GS regimen or other reasons, and 6 patients who met the inclusion criteria withdrew their informed consent prior to treatment. Ultimately, 74 patients were enrolled in this study and available for further analysis (Figure 1). At the final follow-up in September 2020, all but seven censored patients had died and reached a mature survival time.

Baseline characteristics are shown in Table 1. There were no significant differences in age, sex, primary tumor site, metastatic organ sites, blood type, or baseline serum CA19-9 levels between the two groups. The IROX group had a higher proportion of male patients, although the difference was statistically non-significant. Most patients in both groups had elevated serum CA19-9 levels at baseline (IROX: 84%; irinotecan: 80%). Pancreatic head PC occurred in 42.1% (16/38) of patients in the IROX group and 41.7% (15/36) of patients in the irinotecan group. The proportions of patients who previously underwent PC surgery were 63% (IROX) and 66% (irinotecan). In most patients (65%), metastasis was found in only one organ, which was most commonly the liver or abdominal lymph nodes.

**Table 1.** Baseline characteristics of all enrolled patients

Variable	IROX	Irinotecan	*P-*value
Age			
Median (IQR), years	59.5 (55–69.5)	62 (54–64.2)	
≤60 years	16 (42%)	17 (47%)	0.65
>60 years	22 (58%)	19 (53%)	
Sex			
Male	26 (68%)	18 (50%)	0.11
Female	12 (32%)	18 (50%)	
Body mass index			
Median (IQR)	20.2 (18.1–20)	20.2 (18.1–22.1)	
Tumor site			
Pancreatic head	16 (42%)	15 (42%)	0.97
Pancreatic body or tail	22 (58%)	21 (58%)	
Metastatic sites			
Single organ	24 (63%)	24 (67%)	0.85
Multiple organs	14 (37%)	12 (33%)	
Prior surgery			
Performed	24 (63%)	22 (61%)	0.85
Not performed	14 (37%)	14 (39%)	
Baseline CA19-9			
≤37 U/mL	6 (16%)	7 (19%)	0.78
>37 U/mL	32 (84%)	29 (81%)	
Blood type			
A	20 (53%)	17 (47%)	0.81
B	6 (16%)	5 (14%)	
O	10 (31%)	10 (39%)	
AB	2	4	
HBV infection			0.94
Yes	3 (8%)	3 (9%)	
No	35 (92%)	32 (91%)	

IROX, irinotecan plus oxaliplatin; IQR, interquartile range.

### Efficacy and survival

**Figure 2.**
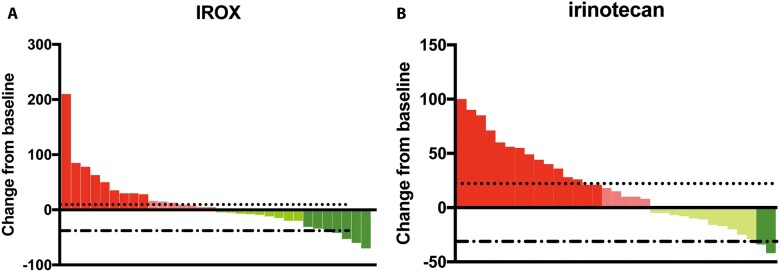
Waterfall plot of the tumor-size change of the IROX group (A) and irinotecan monotherapy group (B). IROX, oxaliplatin plus irinotecan.

The IROX group and the irinotecan monotherapy group received an average of 6.3 and 5.4 cycles of treatment, respectively. The highest numbers of treatment cycles in the IROX group and monotherapy group were 16 and 17 cycles, respectively. Sixty-five out of 74 patients had complete radiologic assessments; the remainder could not be evaluated due to rapid clinical disease progression or other reasons (Figure 2A and B). The ORRs were 18.4% (7/38) and 5.5% (2/36) in the IROX group and irinotecan group, respectively, and the DCRs were 57.9% and 47.2%, respectively. There were no significant differences in the ORR (*P *=* *0.06) or DCR (*P *=* *0.05). A greater proportion of early serum CA19-9 responses was observed in the IROX group than in the irinotecan group, but the difference was non-significant (52.6% vs 44.4%, *P *=* *0.48).

**Figure 3.**
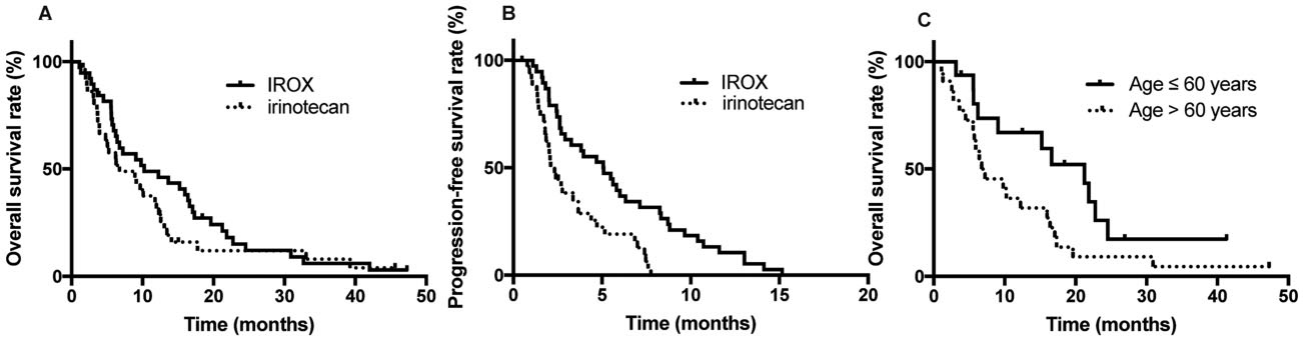
Kaplan–Meier plot for overall survival (A) and progression-free survival (B) according to the treatment arm. Kaplan–Meier plot for (C) IROX group patients according to age (60 years). IROX, oxaliplatin plus irinotecan.

During the follow-up, 67 of the 74 patients died and 7 patients were lost to follow-up. The median survival time was 9.1 months for all patients, 10.2 months (95% CI, 2.2–11.2 months) for patients in the IROX group, and 6.7 months (95% CI, 2.6–17.8 months) for patients in the irinotecan monotherapy group (*P *=* *0.20) (Figure 3A). The median PFS was 5.1 months (95% CI, 2.7–7.4 months) in the IROX group and 2.3 months (95% CI, 1.8–2.8 months) in the irinotecan group (*P < *0.01) (Figure 3B).

**Figure 4.**
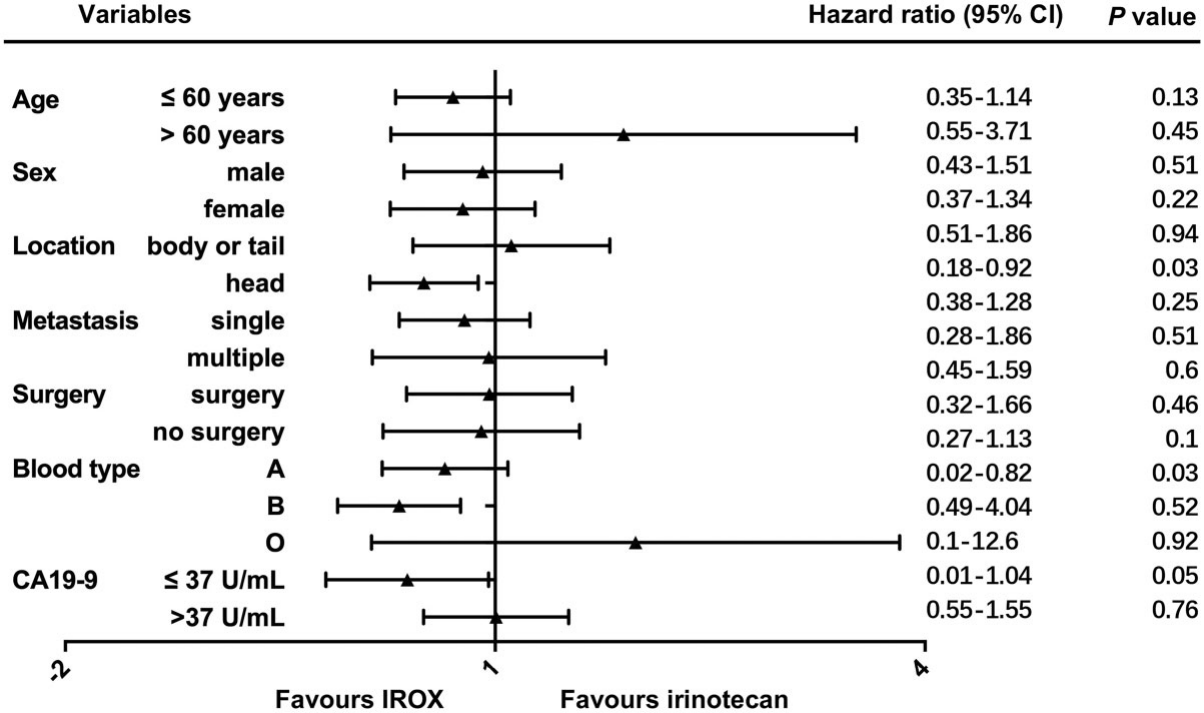
Subgroup analyses of overall survival. CI, confidence interval; IROX, oxaliplatin plus irinotecan.

Early serum CA19-9-responsive patients had an advantageous trend in both PFS (4.4 vs 2.1 months) and OS (10.1 vs 6.2 months). In the irinotecan group, an early serum CA19-9 response was significantly associated with survival (*P *=* *0.07). The OS of primary pancreatic head PC was significantly shorter than that of pancreatic body or tail PC (5.8 vs 10.2 months, *P *=* *0.04). In the irinotecan group, the survival benefit was comparable for patients aged >60 years and younger patients (median overall survival (mOS): 10.1 vs 6.2 months, *P *=* *0.17). In the IROX group, the survival benefit was significantly increased for patients younger than 60 years compared with the patients aged >60 years (mOS: 21.2 months vs 6.7 months, *P *=* *0.04) (Figure 3C). Regarding metastatic organs, the more metastatic organs there were, the worse the prognosis was, but there was no significant difference (mOS: 6.7 vs 10.1 months, *P *=* *0.39). Univariate analysis showed that survival was not significantly associated with prior surgery, blood type, or baseline serum CA19-9 levels in all enrolled patients. With the exception of the older patients (>60 years), the survival benefit of IROX was supported in almost all other conditions (Figure 4).

### Toxicity and safety

Grade 3–4 AEs were observed in 34% (13/38) of patients in the IROX group and 19% (7/36) of patients in the irinotecan group; 23% (9/38) of patients in the IROX group and 11% (4/36) of patients in the irinotecan group underwent dose reduction during treatment. No treatment-related mortality occurred. The most common AE was neutropenia; febrile neutropenia was found in two patients in the IROX group but was not found in any of the patients in the irinotecan monotherapy group. The rates of fatigue, nausea and vomiting, and other non-hematologic toxicities were relatively higher in the IROX group than in the irinotecan monotherapy group (Table 2).

**Table 2:** Grade 3–4 adverse events in two treatment groups

Adverse event	IROX group	Irinotecan group	*P-*value
Hematologic			
Neutropenia	9 (23%)	3 (8%)	0.73
Febrile neutropenia	2 (5%)	0 (0%)	0.16
Thrombocytopenia	6 (15%)	1 (2%)	0.06
Anemia	5 (13%)	3 (8%)	0.50
Non-hematologic			
Fatigue	6 (15%)	3 (8%)	0.32
Vomiting	6 (15%)	4 (11%)	0.55
Nausea	5 (13%)	3 (8%)	0.79
Liver function injury	2 (5%)	1 (2%)	0.59
Peripheral neuropathy	2 (5%)	0 (0%)	0.16
Diarrhea	2 (5%)	1 (2%)	0.59

All values are presented as number of patients followed by percentage in parentheses.

IROX, irinotecan plus oxaliplatin.

## Discussion

This randomized Phase II study evaluated the efficacy, tolerability, and safety of irinotecan monotherapy or irinotecan combined with oxaliplatin as a second-line treatment in mPC patients. The study did not meet its primary study end point. Efficacy and AEs were assessed in all enrolled patients. In this study, second-line treatment, either irinotecan alone or combined with oxaliplatin, prolonged survival in mPC patients. We found that, compared with irinotecan monotherapy, IROX showed a significant advantage in median progression free survival (mPFS) (5.1 vs 2.3 months), and a survival benefit trend in mOS (10.2 vs 6.7 months). Further analysis showed a significant increase in survival benefit of IROX in younger patients (<60 years). The treatment was well tolerated in both groups, and the occurrence rate of Grade 3–4 AEs was acceptable (8%–23%).

Most PC patients are diagnosed at an advanced stage, so systemic chemotherapy plays an important role in the overall management of PC. For second-line treatment, there have been encouraging regiments proven to improve both OS and quality of life [13–15]. The National Comprehensive cancer Network (NCCN) guidelines recommend fluorouracil-based regimens after progression with first-line gemcitabine-based therapy, including FOLFIRI, FOLFOX, and FOLFIRINOX. Second-line FOLFOX or FOLFIRI have a median survival of ∼6 months [16, 17]. FOLFIRINOX extends the median survival to 9.2 months, but it is recommended only for patients with an Eastern Cooperative Oncology Group (ECOG) performance status of 0–1 due to unbearable adverse effects [15]. Therefore, several studies have modified the FOLFIRINOX regimen to reduce its AEs and preserve efficiency. Although various dose-modification regimens exist [18, 19], the regimen without fluorouracil and with only oxaliplatin and irinotecan has not been explored before. In our study, the median OS of patients receiving IROX was 10.2 months, which was slightly longer than that of patients receiving mFOLFIRINOX (mOS, 9.2 months), possibly due to our increased drug-dose intensity (oxaliplatin 85 mg/m^2^, irinotecan 150 mg/m^2^) compared with mFOLFIRINOX (oxaliplatin 65 mg/m^2^, irinotecan 135 mg/m^2^) and the development of support treatment.

Grade 3–4 AEs were more common in the IROX group, with the highest occurrence rate of neutropenia (23% and 8%, respectively). The occurrence rate of AEs in our study was reduced compared with that of mFOLFIRINOX in previous studies, where Grade 3–4 neutropenia accounted for ∼28%–29% of cases [20]. This finding indicates that, after the removal of fluorouracil, IROX may be well tolerated in patients with relatively poor physical states.

Our study still has several limitations. First, it was a single-center Phase II clinical study with a small sample size, and the conclusions need further validation. Second, with the increasing application of gemcitabine plus nab-paclitaxel and FOLFIRINOX, the number of patients who received GS as first-line treatment is decreasing, resulting in a limited number of patients fitting our study-inclusion requirements. Third, the AEs of IROX need to be further verified in future observational studies.

Overall, in this prospective controlled Phase II study, we failed to reach our primary end point. However, IROX showed significantly prolonged PFS over irinotecan monotherapy with acceptable AEs as second-line therapy for mPC. IROX might be an alternative option for some selected patients.

## Authors’ Contributions

All authors contributed to the study conception and design. Material preparation, data collection, and analysis were performed by H.Z., Z.T., and L.L. The first draft of the manuscript was written by H.Z. and all authors commented on previous versions of the manuscript. All authors read and approved the final manuscript.
